# Managing coexisting depression and substance use disorder: insights from clinical experience

**DOI:** 10.3389/fpsyt.2026.1919720

**Published:** 2026-10-28

**Authors:** Fabrizio Schifano, Giovanni Martinotti, Marcin Wojnar, Inês Homem de Melo, Christian Korbel, Gabriele Sani

**Affiliations:** 1 Hertfordshire Medical School, University of Hertfordshire, Hatfield, United Kingdom; 2 Department of Neuroscience, Imaging and Clinical Sciences, G. D’Annunzio University, Chieti, Italy; 3 Department of Psychiatry, Medical University of Warsaw, Warsaw, Poland; 4 CRI Porto Ocidental, Instituto de Comportamentos Aditivos e Dependências (ICAD), Porto, Portugal; 5 Clínica do Quinto Andar, Porto, Portugal; 6 Department for Addiction Diseases, Provincial Clinic Mauer, Mauer, Austria; 7 Department of Neurosciences, Head-Neck and Chest, Section of Psychiatry, Fondazione Policlinico Universitario Agostino Gemelli IRCCS, Rome, Italy; 8 Department of Neurosciences, Section of Psychiatry, Università Cattolica del Sacro Cuore, Rome, Italy

**Keywords:** addiction, comorbidity, depression, MDD (major depressive disorder), substance use & misuse

## Abstract

Comorbid major depressive disorder (MDD) and substance use disorder (SUD) occur frequently and are challenging to manage. The negative impact of these co-occurring conditions on patient quality of life, functional ability, and mortality necessitates timely diagnosis and a multifaceted treatment approach. However, there are few evidence-based clinical guidelines for these co-occurring conditions, further adding to the complexity of treatment. Here, we provide our perspective on the management of MDD in the context of SUD using clinical evidence and our experience. Due to the chronic, relapsing nature of these disorders, long-term follow-up is required to continually assess treatment efficacy and make adjustments where needed. Antidepressants frequently used for MDD, such as selective serotonin reuptake inhibitors (SSRIs), may show variable effectiveness in patients with comorbid SUD, and antidepressant selection should be individualized according to the patient profile, disease characteristics, and type of substance being misused. Treatment options with different pharmacodynamic profiles include agomelatine, bupropion, and vortioxetine. Serotonin antagonist and reuptake inhibitors (SARIs), such as trazodone, may offer advantages in select patients, given their safety profile and potential to address depressive features commonly seen with SUD. Importantly, collaboration between a range of healthcare professionals to provide psychoeducation and pharmacological management, alongside social support that includes family, friends, and/or caregivers is needed for treatment success. Further studies specifically assessing treatments for comorbid MDD and SUD are required. In addition, training is needed to increase medical expertise and lessen stigma, which can contribute to sub-standard care.

## 1 Introduction

Major depressive disorder (MDD) represents a high burden of disease, with a lifetime prevalence of approximately 12% (1). Compounding this, depression and substance use disorder (SUD) frequently co-occur, with an estimated 25% prevalence of SUD among people with MDD (2). Alcohol is the most commonly misused substance in the context of MDD, followed by cannabis and other illicit drugs (2). SUD may present at a similar time or before or after the MDD, and thus the temporal relationship between SUD and MDD may vary and is not uniform (3–5). Regardless of the timing, the association between SUD and MDD is strong (6) and may be underpinned by shared risk factors that include genetic predisposition, neurobiological phenotypes, and environmental variables (7, 8). MDD–SUD presents challenges for treatment, and patients with MDD–SUD are less likely to receive guideline-recommended treatment for depression compared with those with MDD alone (9). In Europe, it is estimated that only 10% of the subset of MDD–SUD patients with alcohol use disorder (AUD), the most common of all SUDs associated with MDD, seek drug treatment, and more than two thirds relapse within the first year of treatment (10). Moreover, treatment-resistant depression (TRD), which is associated with a substantial clinical and psychosocial burden (11), may frequently coexist with AUD or other SUDs, adding to the complexity of MDD–SUD (12).

The World Association on Dual Disorders emphasizes the importance of considering addiction and a co-occurring mental health disorder, such as comorbid MDD–SUD, as one complex condition to enable a more personalized approach with accurate diagnosis and clinical management (13). Indeed, impairment in neurotransmitter systems linked to addictive behaviors and other psychiatric symptoms may be responsible for the shared underlying risks of acquiring comorbid psychiatric disease and SUDs (13). The dopaminergic-mesolimbic system likely has a leading role in the development of addiction and an imbalance in dopamine is also strongly associated with depression and anhedonia (14, 15). Evidence also suggests a complex role for serotonin in regulating a range of neurological functions, including cognition, mood, behavior, and sleep (15). Altered gamma-aminobutyric acid (GABA) and glutamate signaling have also both been linked to addiction, depression, anxiety, and schizophrenia (15). Notably, alcohol affects sleep and wakefulness by interacting with the GABA, serotonin, and glutamate systems (16, 17).

Given that comorbid MDD and SUD adversely affect quality of life (18), neuropsychological functioning (19), and significantly increase the risk of experiencing disability (20), suicidal ideation (21), and mortality (22), it is imperative to accurately diagnose and manage these conditions. Here, we discuss the challenges of comorbid MDD and SUD and draw on our clinical experience to propose best practice management for patients with MDD–SUD.

## 2 Patient presentation

From our perspective, the medical trajectory and timing of comorbid MDD and SUD symptomatology depend on the sequence of MDD and SUD presentation, type of substance, contributing environmental factors, and the underlying neurobiological characteristics of the patient. In turn, the sequence of MDD and SUD presentation can depend on underlying biological, environmental, and behavioral factors (4, 23). For example, the self-medication hypothesis suggests that SUD can develop from use of substances to help cope with symptoms of mood disorders in some individuals (4). The choice of substance used by patients can be based on underlying characteristics and personality, comorbidities (e.g., anxiety), cultural determinants, socioeconomic background, and availability of the substance. Patient scenarios commonly seen in clinical practice are shown in **Figure 1**.

**Figure 1:**
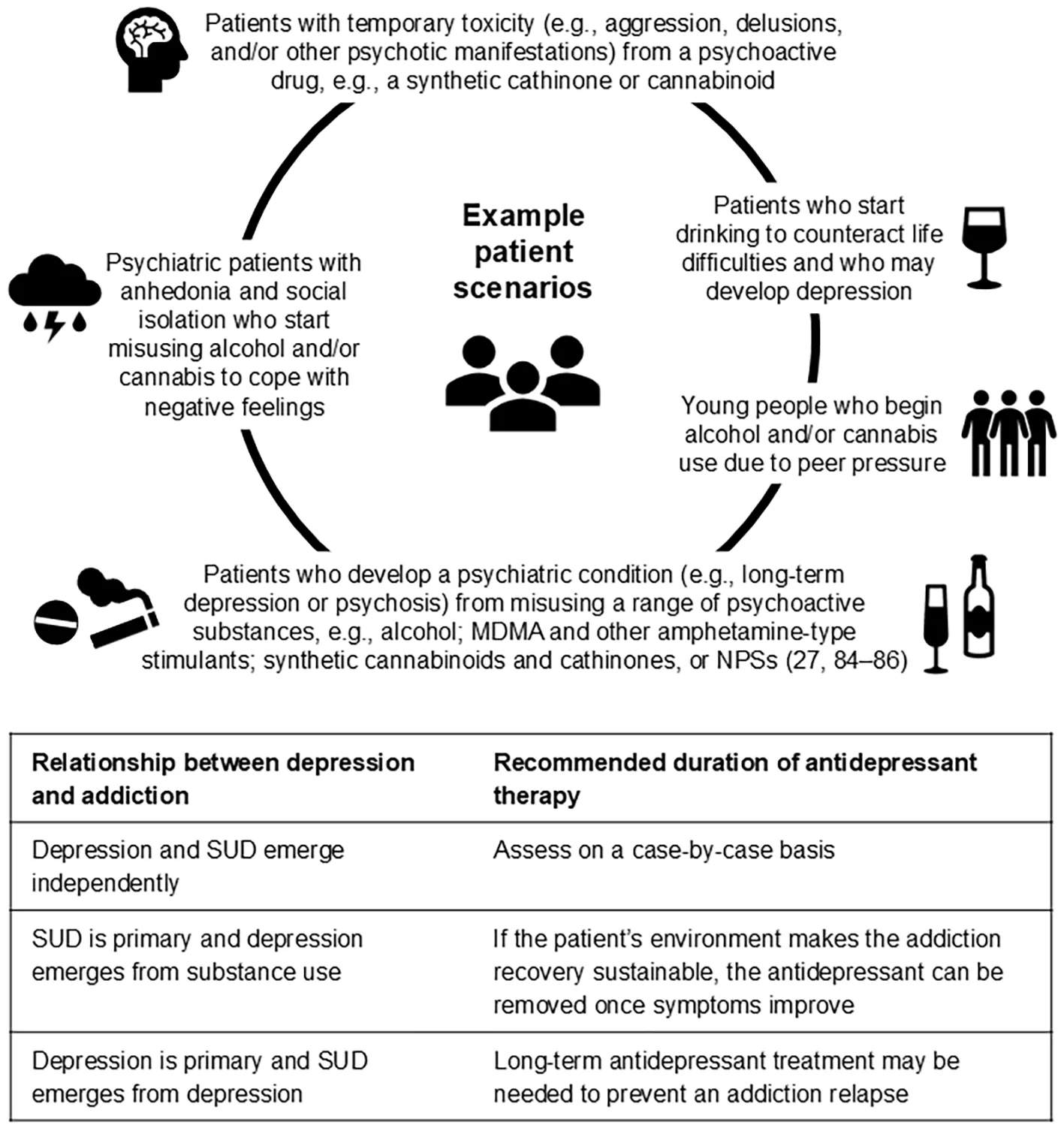
Examples of patient scenarios commonly seen in clinical practice and recommendations for duration of antidepressant therapy depending on the relationship between the depression and the addiction. MDMA, 3,4-methylenedioxymethamphetamine; NPS, novel psychoactive substance; SUD, substance use disorder.

In our experience, SUD is more common in the younger MDD patient population, consistent with the trend for SUD in general (24), with the proportion of SUD likely to be >50% in younger patients with MDD. However, MDD–AUD tends to present higher in middle-aged males alongside symptoms of insomnia and anxiety, although the proportion of females with MDD–AUD is increasing, with the ratio now approximately three males to one female (25, 26). Younger people may use large quantities of alcohol but may not have AUD because it can take time for AUD to develop from regular alcohol use. Young males most frequently use cannabis and novel psychoactive substances (NPSs) (27). Benzodiazepines are most frequently prescribed to females aged 50–64 years for insomnia and/or anxiety, although misuse may occur more commonly in younger adults (28).

True SUD prevalence among patients with MDD is likely to be higher than 25% due to the challenges associated with diagnosing SUD in the context of MDD resulting in missed diagnoses (2, 23). Patient-reported information may underestimate alcohol and drug misuse (29, 30). Moreover, accurate diagnosis is complicated by overlapping symptoms between MDD and SUD, such as apathy, anhedonia, insomnia, psychomotor agitation, and emotional dysregulation, particularly in the context of alcohol or cannabis use where such symptoms are common (31–33). Moreover, substance use can exacerbate these shared symptoms of MDD (34), underscoring the importance of correct diagnosis and management.

## 3 Screening and diagnosis

MDD and SUD are bidirectional and interrelated (35). It is important to assess for both MDD and SUD, regardless of which presents first. Patients with MDD–SUD will have a diagnosis code for MDD and a code for SUD specific to the type of substance, in accordance with the Diagnostic and Statistical Manual of Mental Disorders 5th edition text revision (DSM-5-TR) and International Classification of Diseases (ICD)-10, although ICD-11 has introduced categories for substance-induced mood disorders (36–39). One disorder will often be diagnosed a long time before the other, so there may be a long period of time between assigning the two codes. The order of diagnosis can depend on the setting where the patient first presents. For example, patients in a general psychiatry setting will likely be diagnosed with MDD first and then with the addiction condition later. Conversely, patients in an addiction setting will likely be diagnosed with addiction first, then with MDD. Depending on the substance being misused, it can be challenging to diagnose MDD in a patient with addiction until the patient has been detoxified (34).

In our opinion, protocols and standards are needed to guide general psychiatrists as well as addiction specialists in effective diagnosis and treatment of MDD–SUD. Unified ICD/DSM-5-TR–based screening tools, such as the Beck Depression Inventory (40), Patient Health Questionnaire-9 (41) and Major Depression Inventory for MDD (42) or the DSM-5-TR online assessment measures (36, 43), should be consistently used at all medical settings to ensure neither disorder is overlooked. Routine pretreatment screening for bipolar disorder via clinical assessment and attention deficit hyperactivity disorder (ADHD), using for example the Adult ADHD Self-Report Scale (44) or Diagnostic Interview for ADHD in adults version 5 (45), should also be implemented. Screening for bipolar disorder and ADHD is important to provide an accurate patient profile and appropriate management (46, 47). Presence of ADHD among patients with MDD is common and is associated with more severe MDD episodes and poorer quality of life and functioning (47). Whilst detoxification often takes precedence, simultaneous evaluation for mood disorders is critical to avoid delayed care.

## 4 Management considerations

We recommend a multifaceted approach for management of MDD–SUD, comprising both non-pharmacological and pharmacotherapeutic approaches incorporating dual treatments that can address both the MDD and SUD. Psychiatrists should provide psychoeducation to the patient, including explaining the severity of their condition and the risks associated with continued substance use. Due to having such a chronic, relapsing disease, the patient needs to be prepared for long-term therapy, and the best intervention is the one that the patient will accept and adhere to. It is important to involve a team of psychiatrists, psychologists, addiction therapists, psychotherapists, and primary care physicians (PCPs) as well as family members, friends, and caregivers to work together and support the patient. Family support groups are important, as family dynamics and relationships can contribute to the propensity for addiction and depression relapses; indeed, engaging family members in psychoeducation can reduce the risk of relapse (48). Structured, center-based groups focused on relapse prevention and peer-support groups can be particularly valuable for psychosocial support.

The underlying cause of MDD (primary or substance-induced) and type of substance is important for treatment decisions. Psychiatrists must understand the characteristics of the specific substance, including the effects of withdrawal and possible drug–drug interactions when developing a treatment plan. The addiction often needs to be treated first, because treatment for MDD may not be effective in the presence of substance misuse. Varying levels of detoxification are needed before antidepressant treatment can start, depending on the substance in question. For example, treatment of opioid addiction with opioid agonist therapy can take years, so the MDD needs to be treated at the same time. Conversely, 7–10 days can be a sufficient detoxification period to become alcohol-free and overcome withdrawal symptoms of AUD, or antidepressant medication can be started whilst trying to reduce alcohol intake. The psychiatrist should explain to the patient the potential effects, risks, benefits, and implications of each therapeutic option, and collaborate with the patient in shared decision-making.

## 5 Treatment of MDD–SUD

A summary of our recommendations for treatment of MDD–SUD is provided in **Table 1**. Overall, combination strategies that address each condition should be considered (49). In our opinion, comorbid MDD and SUD typically require a longer duration of treatment than for either disorder alone, particularly in cases of severe addiction or where there is a risk of suicide, since MDD relapses are the norm and not the exception alongside addiction. Full recovery from MDD is the aim, but the result at times may only be reducing its severity. Regular follow-up is essential, and re-evaluating the treatment decisions with the patient after 6 months is recommended. Longer-term antidepressant treatment is often required but this duration may be seen by patients as difficult to accept initially, because patients may need to adjust to having a potentially chronic condition that may not resolve quickly. Patients also need to overcome the perceived stigma of using antidepressants on a long-term basis (50). The exact duration of the antidepressant therapy can depend on the interconnectivity between the MDD and the SUD, i.e., longer-term antidepressant treatment may be required to prevent an addiction relapse if the SUD was developed as a coping mechanism to the MDD (**Figure 1**). Harm-reduction strategies are foundational and may be prioritized, particularly in older patients with a prolonged history of depression, substance use, and multiple relapses.

**Table 1:** Recommendations for treatment strategies by type of substance use disorder.

Diagnosis	SUD treatment	Depression treatment	Practical considerations
MDD with alcohol use disorder	• Detoxification with benzodiazepine (e.g., diazepam or lorazepam/ oxazepam) • Medication for relapse prevention as needed, such as opioid antagonists (e.g., nalmefene, naltrexone), or GABA analogues (e.g., acamprosate)	• Options include trazodone, mirtazapine, vortioxetine, clomipramine • Mood stabilizers or atypical antipsychotics (e.g., aripiprazole, quetiapine or cariprazine) may be useful for psychotic symptoms or bipolar features, including severe agitation and/or affective instability	• Start with monotherapy, add other therapies as needed • Antidepressants with sedative properties are preferable (to avoid benzodiazepine use) • Lithium may be prescribed to help reduce aggressive or self-harming behaviors; olanzapine may be prescribed to help manage psychotic symptoms, if needed • Be aware of potential seizures during withdrawal
MDD with cannabis use disorder	• Gradual THC taper or supervised cessation	• Options include trazodone, mirtazapine, vortioxetine, TCAs (clomipramine), bupropion + trazodone • Sleep adjuncts (melatonin or promethazine) can also help • DPRAs (aripiprazole, cariprazine) or other atypical antipsychotics (amisulpride) can be used for psychosis mitigation	• Patients tend to be younger, so this needs to be taken into consideration with treatment choices (e.g., they may prefer medication without sexual side effects) • Firstly, it should be determined if the patient has any symptoms of psychosis from cannabis use • Combination therapy is often used to treat the depression • Prescribe lithium or clozapine for harm reduction, if needed • Motivational interview techniques and a systemic approach (e.g., including family members) is particularly useful
MDD with benzodiazepine use disorder	• Introduce long-half-life benzodiazepines, then eventually decrease the amount	• Options include trazodone, mirtazapine, TCAs (clomipramine), gabapentinoids (pregabalin), SSRIs (paroxetine, escitalopram), vortioxetine • Oxazepam can substitute for the benzodiazepine the patient is addicted to and be tapered down with time • Combinations can be effective, e.g., paroxetine or escitalopram + trazodone or mirtazapine + mood stabilizer • Atypical antipsychotics (quetiapine) may be useful as an adjunctive treatment	• MDD with benzodiazepine use disorder is more common among older adult males, and is less frequent among adolescents or young adults • Managing benzodiazepine addiction is a long process, involving a long, protracted withdrawal, meaning that waiting for detoxification before starting antidepressants is not feasible • Co-occurring anxiety and depression are common • Combination therapy is most often used to treat the depression • Avoid bupropion due to potential seizure risk during withdrawal
MDD with cocaine/ stimulant use disorder	• Brain stimulation strategies (e.g., TMS); possibly prescription stimulants (methylphenidate, dexamphetamine, lisdexamfetamine, amphetamine salts, bupropion)	• Options include dopaminergic antidepressants (e.g., bupropion), DRPA atypical antipsychotics (e.g., cariprazine, aripiprazole), SARIs (e.g., trazodone) • Bupropion can be combined with a DRPA atypical antipsychotic, or methylphenidate/ dexamphetamine in the presence of ADHD, if needed	• Further information on NPSs is needed before any recommendations can be given on these substances • Depression usually starts when patients stop the substance use • There is a lack of viable treatment options for cocaine/stimulant use disorder and depression • Depression in these patients can be long term and is difficult to treat; combination therapy is most often used • A higher dosage of antidepressant treatment may be required as receptors may have become insensitive from cocaine/stimulant use

ADHD, attention deficit hyperactivity disorder; DRPA, dopamine receptor partial agonist; MDD, major depressive disorder; NPS, novel psychoactive substance; SARI, serotonin antagonist and reuptake inhibitor; SSRI, selective serotonin reuptake inhibitor; SUD, substance use disorder; TCA, tricyclic antidepressant; THC, tetrahydrocannabinol; TMS, transcranial magnetic stimulation.

The choice of the initial antidepressant depends on both a patient’s individual symptoms and co-occurring conditions (e.g., sleep disturbances, anxiety, bipolar disorder, or ADHD) and clinical setting (inpatient or outpatient) (35). Monotherapy may be considered initially, but combination therapy is often required in our experience. Historically, the evidence supporting use of antidepressants in MDD–SUD is low quality (51), although data suggest antidepressants may be useful for the treatment of depression, alcohol dependence, or both, in people with MDD–AUD (52). A meta-analysis from 2005 found that most studies showed a significant improvement of depressive scores in patients with AUD receiving an antidepressant versus placebo, except for selective serotonin reuptake inhibitors (SSRIs) (53). The heterogeneity of the available evidence may be a possible explanation for the absence of a significant effect seen with SSRIs (53). It could also be speculated that a limited ability to improve symptoms of anhedonia and a tendency to induce emotional blunting, as widely reported in the literature for SSRIs and serotonin-norepinephrine reuptake inhibitors (SNRIs), could affect the results (54–59). Anhedonia itself is a key symptom of depression, often contributing to substance use relapse (60). While SNRIs and norepinephrine reuptake inhibitors (NRIs) may not be effective on the addiction-related dimension of MDD–SUD, some evidence suggests favorable outcomes in patients with cocaine addiction (61, 62).

Trazodone, a serotonin antagonist and reuptake inhibitor (SARI), is an antidepressant approved for the treatment of MDD; it is characterized by a multimodal pharmacological profile (63). Trazodone interacts with serotonin receptors and transporters in a way that modulates neuronal activity through multiple neurotransmitter systems, including the serotoninergic, dopaminergic, adrenergic, and histaminergic systems (63–65). Through these interactions, trazodone induces a fast antidepressant response and has sedative effects (63–65). Therefore, trazodone may represent a useful option in select patients with MDD and SUD because it can also relieve the MDD symptoms of anxiety, agitation, and insomnia that are also common in patients with SUD and may be linked to early relapse (17, 34, 66–69). Trazodone has demonstrated antidepressant effectiveness in MDD–AUD and has been reported to help reduce craving for alcohol (66, 70). Trazodone is not generally associated with an addiction liability (68) and preliminary observations support further exploration of possible anti-substance-craving properties (71). Additionally, the different formulations of trazodone allow it to be used in different phases of the treatment strategy (63, 64). Trazodone has a favorable safety and tolerability profile, which is particularly important for young users who do not wish to experience weight gain or sexual dysfunction, and for longer term use (67, 72). Moreover, SARIs may be associated with less emotional blunting than SSRIs and SNRIs (58, 73, 74).

Other treatment options with different pharmacodynamic profiles that can be considered based on our clinical experience include: agomelatine, a melatonin agonist and serotonin antagonist antidepressant with possible anti-craving properties (75–77); vortioxetine, a serotonin modulator and stimulator with preliminary evidence in patients with MDD–AUD (78); and bupropion, a selective inhibitor of norepinephrine and dopamine reuptake with minimal effects on serotonin activity (79), which can be useful for treating MDD in the context of SUD (80). These aforementioned agents are generally associated with favorable anti-anhedonic properties (81). Medications for SUD, such as naltrexone for AUD, can be combined with antidepressants to improve treatment efficacy (82, 83). In cases of major depressive episodes with psychotic features co-occurring with AUD or SUD (84–86), specific recommendations should be followed; current evidence suggests a preference for long-acting antipsychotics and dopamine receptor partial agonists (87). Based on our experience, psychoeducation and social support are also critical alongside pharmacotherapy, and a collaborative support network involving family and caregivers should be established. Cognitive behavioral therapy (CBT) or other types of psychotherapy should also be recommended to each patient for both addiction and MDD.

## 6 Discussion

The incidence of mental health disorders has increased with time, particularly among adolescents and young adults (88). Together with the increasing potency and addictive properties of illicit substances in recent years (89–91), we expect to see a rise in the frequency of comorbid psychiatric disease and SUD. Appropriate treatment of MDD–SUD is critical to help relieve the impact of this challenging disorder on patients’ lives. However, the use of antidepressant treatment in MDD–SUD is suboptimal (9, 10). A key treatment challenge is that there are few guidelines for comorbid psychiatric disease and SUD (92), and so healthcare professionals (HCPs) rely heavily on clinical experience. Here, we have drawn upon our professional experience to summarize management strategies for the clinical community (**Table 1**). In our opinion, professionals need to organize follow ups with patients with MDD–SUD over a long period of time, and a multidisciplinary approach should be the golden standard of care. Ideally, multidisciplinary teams should be led by psychiatrists adequately trained in addiction, and can include PCPs, psychotherapists, psychologists, addiction therapists, and social workers. CBT and other psychotherapeutic approaches should be recommended as part of the treatment for each patient to treat both the SUD and MDD. In the future, increased use of virtual/digital tools that are available 7 days a week, 24 hours a day, will be needed to enhance MDD–SUD care.

There are many challenges to address for best practice management of comorbid psychiatric disease and SUD. While experts have published recommendations (93, 94), more evidence-based guidelines are required to support management decisions. Moreover, additional clinical trials in MDD–SUD are necessary to help build the body of evidence regarding the differential benefits of various treatments for various patient profiles and scenarios and to evaluate novel emerging treatments. Recent evidence suggests that glucagon-like peptide 1 receptor agonists (GLP-1 RAs) may help alleviate addictive behaviors, including drug and alcohol misuse (95, 96). A Phase II randomized clinical trial in AUD has provided preliminary evidence that the GLP-1 RA semaglutide can reduce craving and some drinking outcomes (97). In addition, greater availability of specific training for addiction psychiatry is needed. Protocols for diagnosis and treatment of comorbid MDD and SUD that do not depend on the setting in which the patient first presents would be extremely valuable. Additional units, teams and/or programs specializing in comorbid psychiatric disease and SUD would also improve care. Integrated facilities for addiction and general psychiatry could enable more appropriate and timely diagnosis of MDD–SUD and prevent “first-seen” bias, whereby SUD can go undetected in a psychiatric ward and MDD can be overlooked in an addiction setting. Diagnostic overshadowing is also an issue for patients with mental health disorders and/or addiction, leading to mental health conditions not being recognized.

Stigma presents a challenge for effective diagnosis and management of MDD–SUD by discouraging open discussion and contributing to fragmented and inadequate care. Stigma exists at every level: within general psychiatry, primary care, and wider society (98, 99). There can also be stigmatization if HCPs attribute a patient’s symptoms to addiction without considering concomitant mental health disorders. Additional training of HCPs is important for reducing stigma around addiction and highlighting the treatability of psychiatric disease. Service structures can also present barriers to obtaining optimal treatment prescriptions for comorbid psychiatric disease and SUD, with insufficient training of addiction psychiatrists in some countries (100, 101). Furthermore, addiction service staff, at least in some countries, are often not clinically trained psychiatrists or psychologists. Nurses and pharmacists are only allowed to prescribe medications in certain countries and are unlikely to be qualified to make a diagnosis of MDD–SUD, resulting in suboptimal care for patients. PCPs have not always received adequate training in the different treatment options available and may not be confident or able to prescribe what the psychiatrist recommends. Compounding this, demand for addiction services is high and mental health units are often oversubscribed and may not accept patients with comorbid psychiatric disease and SUD if symptoms are attributed purely to addiction. Overall, additional training of HCPs and exposure to addiction services to increase medical expertise is critical.

## 7 Conclusion

MDD–SUD impacts every aspect of a patient’s life and people with comorbid MDD and SUD deserve integrated treatment. Multiple treatment options are available for MDD–SUD, and a personalized approach is vital. While SSRIs are commonly prescribed for patients with MDD, evidence suggests that they may not always be optimal options for some patients with MDD–SUD (53). Therefore, other antidepressants must be regarded. Trazodone is an antidepressant that may represent a useful treatment option for MDD in the context of SUD. Trazodone has a favorable safety and tolerability profile, and is unlikely to be limited by emotional blunting. Challenges beyond antidepressant choice also exist for optimal management of comorbid psychiatric disease and SUD, and further research and improvement of medical training, facilities, and practices are required.

## Data Availability

The original contributions presented in the study are included in the article/supplementary material. Further inquiries can be directed to the corresponding author.
